# The Past, Present, and Future of Sentinel Lymph Node Biopsy in Breast Cancer

**DOI:** 10.1245/s10434-026-19672-4

**Published:** 2026-05-29

**Authors:** Michael Stanczyk, Tiffany C. Cheung, Julie E. Lang

**Affiliations:** 1 Cancer Biology, Cleveland Clinic, Lerner College of Medicine, Case Western Reserve University School of Medicine, Cleveland, OH USA; 2 Department of Breast Services, Cleveland Clinic, Cleveland, OH USA; 3 Division of Surgical Oncology, University of Kentucky, Lexington, KY USA

**Keywords:** Sentinel Lymph Node Biopsy, Targeted Axillary Dissection, Breast cancer, Axillary staging, Neoadjuvant chemotherapy, Axillary Lymph Node dissection, Lymphatic mapping

## Abstract

Sentinel lymph node biopsy (SLNB), the current gold standard for axillary staging for many breast cancer patients, has replaced axillary lymph node dissection (ALND) for clinically node-negative patients due to lower surgical morbidity while maintaining oncologic safety. Advances in breast cancer surgery, axillary staging, lymphatic mapping, targeted axillary dissection (TAD), neoadjuvant chemotherapy (NAC), and sentinel lymph node (SLN) identification techniques have reshaped axillary management and enabled progressive de-escalation of surgical treatment. This review examines the historical development, current clinical applications, and emerging innovations in SLNB. Early randomized trials including the NSABP B-32 and ALMANAC trials established SLNB as a safe alternative to ALND for clinically node-negative patients with comparable overall survival (OS), disease-free survival (DFS), and regional control while significantly reducing complications such as lymphedema. Subsequent trials including ACOSOG Z0011, IBCSG 23-01, and AMAROS further supported omission of completion ALND (cALND) for selected patients with limited SLN metastases. In the neoadjuvant setting, studies such as ACOSOG Z1071, SENTINA, and SN-FNAC evaluated SLNB accuracy after NAC and informed the development of optimized techniques, including dual-tracer mapping and TAD, to reduce false-negative rates. Technological advances including indocyanine green fluorescence imaging, technetium-99m radiotracers, and superparamagnetic iron oxide tracers have further improved SLN identification. Ongoing trials investigating SLNB omission and imaging-guided axillary staging continue to refine patient selection and advance the shift toward personalized, morbidity-conscious axillary management.

Sentinel lymph node biopsy (SLNB) is the gold standard for many breast cancer patients.^1^ The procedure identifies and removes the first draining (sentinel) lymph node or nodes to determine the presence of metastasis. As a minimally invasive alternative to complete axillary lymph node dissection, SLNB reduces surgical morbidity while providing the pathologic information needed to guide further axillary management and adjuvant therapy.

This review examines the development of axillary SLNB, its current clinical applications, and opportunities for improvement. Because precision surgery and morbidity reduction remain priorities in breast cancer care, reassessing the evolving role of SLNB is increasingly important. By examining the origins and evolution of SLNB, we can gain a deeper understanding of its significance in modern breast cancer treatment and how advancements can further refine the procedure.

## Past

### Historical Background of Axillary Surgery

The origins of axillary surgery trace back to Dr. William Halsted’s radical mastectomy, which removed the entire breast, overlying skin, pectoral muscles, and axillary lymph nodes.^2^ The Halstedian paradigm held that increasingly extensive surgery improved local control and survival in an era without systemic therapy. After the introduction of adjuvant chemotherapy and endocrine therapy in the 1970s and 1980s, they quickly became standard treatments, particularly for node-positive breast cancer patients, significantly contributing to the reduced reliance on extensive surgery.

The Fisher paradigm emphasized the importance of systemic therapy over radical local control.^3^ This shift was most clearly demonstrated in the NSABP B-04 trial that compared radical mastectomy with total mastectomy without axillary lymph node evaluation, with or without regional radiation in clinically node-negative (cN**–**) patients. Importantly, B-04 showed no survival advantage over radical mastectomy despite complete omission of nodal assessment, providing the first randomized evidence that routine axillary clearance was not required for disease control.^4^ These findings established axillary lymph node involvement as a prognostic rather than a therapeutic determinant.

Axillary lymph node dissection (ALND) carries significant morbidity, most notably lymphedema. A 20-year longitudinal study found that the cumulative lymphedema rates after ALND ranged from 13 % to 48 %, depending on definition and timing.^5^ Early lymphedema may occur within weeks to months after surgery, whereas late-onset lymphedema can develop years later. The extent of nodal removal is a major predictor of lymphedema, with patients who undergo dissection of more than 10 lymph nodes experiencing substantially higher rates.^6^

Long-term complications and a growing understanding of tumor biology prompted a re-evaluation of ALND’s role, ultimately paving the way for the development of SLNB.

### Introduction of SLNB

As systemic therapy evolved, the routine need for ALND, long considered the standard approach to axillary staging, began to be questioned, particularly for elderly patients and those with small, early-stage tumors.^7–10^ Veronesi et al.^11^ demonstrated that axillary lymphatic spread followed a sequential pattern in which level 1 nodes were almost always involved before higher levels, supporting the concept of selective nodal sampling. In 1994, Dr. Giuliano introduced the SLNB for breast cancer, adapting a technique previously used in melanoma, and found that SLNB was 95.6 % as accurate as a complete ALND in correctly predicting the axillary nodal status.^12^ Veronesi et al.^13^ conducted a randomized trial to compare SLNB alone and routine ALND in cN- patients with tumors smaller than 2 cm. The trial found no significant difference in overall survival (OS) or disease-free survival (DFS) between the groups, providing evidence that SLNB could safely replace ALND for patients with early-stage, cN– breast cancer.

Meta-analyses have confirmed the high accuracy of SLNB, reporting a pooled identification rate of 96 %, a sensitivity of 93 %, an overall accuracy of 99 %, and a false-negative rate (FNR) of 5 % to 6 %.^14^ These outcomes demonstrate that SLNB reflects the status of the axilla in 97 % of cases.^15^ However, long-term studies were needed to confirm that less invasive approaches offer non-inferior OS and DFS and that comorbidity is significantly decreased.

In addition to validating SLNB in cN– patients, further refinements were developed to maintain accuracy in more complex settings. Targeted axillary dissection (TAD), which combines SLNB with retrieval of a previously clipped node that was biopsy-proven metastatic before neoadjuvant chemotherapy (NAC), is a strategy to reduce FNR in clinically node-positive (cN+) patients. Because the clipped node is not consistently identified as a sentinel lymph node (SLN), occurring in approximately 23 % of cases, omission of its retrieval may contribute to staging inaccuracy. By combining anatomic targeting with lymphatic mapping, FNR was reduced to 1.4 % in the prospective study by Caudle et al.^16^ that described TAD.

### Key Landmark Trials

From the early 2000s onward, a series of randomized controlled trials established SLNB as an effective alternative to ALND for axillary staging of selected patients. Across multiple clinical settings, these trials demonstrated comparable OS, DFS, and regional control while consistently reducing surgical morbidity and improving quality of life.

The Axillary Lymphatic Mapping Against Nodal Axillary Clearance (ALMANAC) trial compared SLNB with ALND among patients with operable cN– breast cancer. Patients undergoing primary breast surgery were randomized to SLNB alone or conventional ALND. This trial demonstrated that SLNB was associated with significantly lower rates of lymphedema, sensory disturbance, and shoulder dysfunction. Additionally, patient-reported quality of life was significantly higher, and axillary control was not compromised.^17^

The National Surgical Adjuvant Breast and Bowel Project B-32 (NSABP B-32) assigned patients to SLNB and ALND (group 1) or to SLNB then ALND if the SLNB exhibited a positive sentinel lymph node (group 2). The 8-year Kaplan-Meier estimates were 91.8 % and 90.3 % for OS and 82.4 % and 81.5 % for DFS for groups 1 and 2, respectively.^18^ The SLNB procedure was non-inferior in DFS and OS and demonstrated significantly lower morbidity, including reduced arm volume changes, sensory symptoms, and shoulder dysfunction.^19^ These findings established SLNB as an effective and less morbid alternative to ALND for cN–patients.

The American College of Surgeons Oncology Group Z0011 (ACOSOG Z0011) trial randomized early-stage breast cancer patients treated with breast-conserving surgery (BCS) with one or two positive SLNs to SLNB or ALND. During a median follow-up period of 9.3 years, OS and DFS were found to be non-inferior to SLNB alone, and rates of locoregional recurrence were similar (6.2 % vs 5.7 %). Notably, ACOSOG Z0011 excluded patients with positive palpable nodes, more than two positive SLNs, omission of whole-breast irradiation, mastectomy without radiation, or receipt of NAC.^20^ The trial did not require axillary ultrasound. A critique of the trial was the lack of detailed radiation plans, raising the concern that some local control benefit could be due to low axillary radiation tangents.^21^ Overall, this trial supported SLNB for BCS patients who had no more than one or two positive nodes without compromising long-term outcomes.

The Asociación Andaluza de Tratamiento Radical Modificado (AATRM) 048/13/2000 trial evaluated the role of completion ALND (cALND) after SLNB for early breast cancer patients with SLN micrometastasis (<2 mm). Patients with cN– underwent SLNB, and those found to have SLN micrometastasis were randomized to cALND or observation. During a median follow-up period of approximately 5 years, the DFS observed between groups did not differ significantly. These findings support omission of cALND for patients with low-volume nodal disease identified by SLNB.^22^

The ACOSOG Z1071 trial evaluated the accuracy of SLNB for cN+ patients after NAC. The overall FNR was 12.6 %, exceeding the accepted 10 % threshold, but improved to 10.8 % with dual tracer and decreased further when two or more SLNs were removed.^23^ Although this was a negative trial, clinical practice evolved to accept SLNB after NAC. Two schools of thought emerged in which previously positive lymph nodes were marked with a clip or conventional SLNB was performed without targeting a clipped node. Targeted axillary dissection was subsequently shown to reduce FNR to approximately 2 % to 4 %, whereas optimized SLNB with dual tracers and retrieval of three or more nodes achieved FNRs below 10 %.^16,24^ These differing approaches ultimately shaped current guideline recommendations and institutional practice patterns regarding post-NAC axillary staging.

The Sentinel Neoadjuvant (SENTINA) trial assigned patients to one of four arms: arm A included cN- patients undergoing SLNB before NAC; arm B consisted of patients who were SLN-positive before NAC and underwent repeat SLNB after NAC; arm C consisted of cN+ patients who converted to cN– disease after NAC and underwent SLNB followed by ALND; and arm D consisted of patients who remained node-positive after NAC and directly proceeded to ALND. The trial found that the patients in arm C had an FNR of 14.2 % and an SLN identification rate of 80.1 %. However, improvements in techniques, including the use of blue dye, increased the detection rate to 87.8 %, and the combination of blue dye and radiocolloid decreased the FNR to 10.8 %.^25^ Although the initial FNR raised concerns about SLNB after NAC, the impact of technique suggested that accuracy could improve with optimized mapping.

The Sentinel Node Biopsy Following Fine-Needle Aspiration Cytology (SN-FNAC) trial evaluated the accuracy of SLNB after NAC for cN+ patients. The patients underwent SLNB followed by ALND with routine use of immunohistochemistry (IHC) to enhance detection of residual nodal disease. The SLN identification rate was 87.6 %, and the FNR was 8.4 %.^26^ This trial demonstrated that optimized SLNB techniques can achieve clinically acceptable accuracy after NAC for selected cN+ patients.

The T1–T2 Breast Cancer: Comparison Between Removal and Preservation of Axillary Lymph Nodes in the Presence of Sentinel Lymph Node Metastases (SINODAR ONE) prospective multicenter trial evaluated whether ALND could be safely omitted for patients with T1–T2 breast cancer and one or two macrometastatic SLNs. Patients were randomized to ALND with adjuvant therapy or to SLNB alone with adjuvant therapy. At the 3-year follow-up evaluation, recurrence-free survival (RFS) and OS were nearly identical between the study arms. These findings support de-escalation of axillary surgery for early-stage patients with limited SLN involvement.^27^

The Sentinel Node vs Mastectomy/Axillary Clearance (SENOMAC) trial evaluated omission of ALND for patients with T1–T3 breast cancer and one or two macrometastatic SLNs. Candidates for BCS and mastectomy were randomized to ALND or no further axillary surgery after SLNB. Omission of ALND did not compromise DFS, supporting the safety of axillary surgery de-escalation for patients with limited SLN macrometastases beyond the ACOSOG Z0011 patient population.^28^

The Neoadjuvant Sentinel Lymph Node Biopsy in Turkey (NEOSENTI-TURK MF18) trial assessed the feasibility of SLNB or TAD with adjuvant radiation for cN+ patients who achieved nodal pathologic complete response (pCR) after NAC. The trial demonstrated that ALND was unnecessary for patients with limited residual nodal burden across all biologic subtypes, thereby expanding the inclusion criteria to include patients that can safely omit ALND.^29^

The International Breast Cancer Study Group (IBCSG) 23-01 trial further supported omission of ALND for patients with limited SLN disease. This multicenter non-inferiority trial randomized cN– patients with SLN micrometastasis (<2 mm) found after SLNB were randomized to ALND or no further axillary surgery. The 5-year DFS was 87.8 % without further axillary surgery and 84.4 % with ALND, demonstrating that even patients with minimal nodal involvement could safely avoid ALND.^30^

The Axillary Management: Axillary Radiotherapy or Surgery (AMAROS) trial evaluated axillary radiation as an alternative for node-positive patients after SLNB, randomizing them to axillary radiation or ALND (1166 patients [82 %] were treated by BCS, and 248 patients [18 %] were treated by mastectomy). The groups did not differ significantly in OS, DFS, or 5-year axillary recurrence rate (1.19 % vs 0.43 %). Axillary radiation, however, resulted in significantly lower incidence and severity of upper extremity lymphedema.^31^ Long-term follow-up evaluation at 10 years confirmed these findings: axillary recurrence did not differ significantly (1.82 % with axillary radiation vs 0.93 % with ALND), and OS and DFS were comparable.^32^

The Optimal Treatment of the Axilla–Surgery or Radiotherapy (OTOASOR) trial was a randomized, phase 3 study comparing complete ALND with regional nodal irradiation in cN– patients. Patients with a positive SLN found during SLNB were randomized to ALND or regional nodal irradiation after BCS. The groups did not differ significantly in DFS, OS, or axillary recurrence, and treatment-related morbidity was reduced, supporting irradiation as an effective and less morbid alternative for patients with limited nodal disease.^33^

The Intergroup Sentinel Mamma Study (INSEMA) trial evaluated omission of SLNB for early breast cancer cN– patients. The 5-year DFS was similar between the groups (91.7 % with SLNB vs 91.9 % without SLNB), reinforcing that axillary staging may not be necessary for hormone-positive, human epidermal growth factor receptor 2 (HER2)-negative low-risk patients. Although patients who omitted SLNB had a slightly higher axillary recurrence incidence (1.0 % vs 0.3 %), they experienced fewer deaths (1.4 % vs 2.4 %) and reported significantly better quality of life.^34^ Reflecting these findings, the 2025 American Society of Clinical Oncology (ASCO) guidelines currently support SLNB omission for postmenopausal, cN– patients older than 50 years with T1–T2, low-risk hormone receptor-positive, HER2-negative tumors treated with BCS and whole-breast irradiation.^35^

The SOUND trial randomized 1405 early-stage cT1N0 patients with planned BCS and radiation to SLNB or omission of SLNB. The OS and 5-year DFS were non-inferior in the omission group (approximately 94 % to 98 % in both groups).^36^These findings highlight that although SLNB is less morbid than ALND, it still carries potential long-term complications and may be unnecessary for low-risk patients. These findings provide evidence that it is safe to de-escalate care by avoiding unnecessary surgery in low-risk cases.

The multicenter Oncoplastic Breast Consortium-05/ICARO cohort study retrospectively evaluated 583 patients with isolated tumor cells (ITCs) in SLNs after NAC, comparing cALND with no further axillary surgery. Completion ALND yielded minimal additional nodal disease and did not improve oncologic outcomes, with a 3-year axillary recurrence rate of 2 % and an invasive recurrence rate of 11 % regardless of surgical approach.^37^ These findings suggest that routine ALND is not supported with ypN0i+ disease, supporting further de-escalation of axillary surgery for this population in which management has previously been unclear, although longer follow-up evaluation remains warranted.

The National Surgical Adjuvant Breast and Bowel Project (NSABP) B-51/RTOG 1304 trial evaluated whether regional nodal irradiation is necessary for cN+ patients who achieve pCR after NAC. After surgery, patients were randomized to whole-breast irradiation with or without regional nodal irradiation. No significant difference in DFS was observed between the treatment arms, indicating that omission of regional nodal irradiation did not compromise oncologic outcomes for this population. These results support a response-based approach to post-NAC treatment.^38^

The Sentinel Node Omission Guided by Axillary PET Imaging (SOAPET) trial is a phase 2 study evaluating whether axillary positron emission tomography (PET) (LymphPET) can improve preoperative axillary assessment sufficiently to permit omission of SLNB for cN– patients. In the first stage, the negative predictive value of LymphPET for nodal macrometastasis was 87.8 %, and when combined with ultrasound, increased to 91 %. These findings support the feasibility of an imaging-guided approach to axillary management and provide a rationale for ongoing investigation into whether optimized preoperative imaging can safely identify patients for whom SLNB may be omitted.^39^

These landmark trials demonstrated that in many cases, SLNB is a suitable alternative to ALND for axillary staging (Table 1). Building on this foundation, subsequent investigations evaluated whether further axillary surgery could be omitted or replaced with regional nodal irradiation for carefully selected patients (Table 2). Although SLNB has transformed early-stage breast cancer management, its role continues to evolve as new evidence and emerging technologies refine patient selections and surgical technique. Currently, SLNB remains a central component of axillary management and continues to be optimized to improve patient outcomes (Table 3).

**Table 1:** Published trials comparing ALND and SLNB

Trial	Year	Initial treatment approach	Node status	Inclusion criteria	Key findings
ALMANAC	2006	Upfront surgery	cN–	Primary invasive breast cancer and cN–	SLNB non-inferior to ALND for axillary local recurrence with reduced lymphedema and complications
NSABP B-32	2010	Upfront surgery	cN–	Primary invasive breast cancer and cN–	SLNB non-inferior to ALND for overall survival and disease-free survival with less morbidity
ACOSOG Z0011	2011	Upfront surgery	cN– → pN1	Invasive breast carcinoma clinically 5 cm or less and cN–	SLNB non-inferior to ALND for overall survival
AATRM 048/13/2000	2012	Upfront surgery	cN– → micrometastasis (<2 mm)	Early-stage cN– breast cancer	SLNB non-inferior to ALND for disease-free survival for patients with micrometastasis
ACOSOG Z1071	2013	NAC	cN+	T1-T4, N1–N2, M0 primary invasive breast cancer	Use of dual-agent mapping and recovery of more than 2 SLNs lowered FNR to 10.8 %
SENTINA	2013	NAC	cN– cN– → pN1 cN+ → ycN0 cN+ → ycN1	Early-stage breast cancer undergoing NAC	Post-NAC SLNB in cN+ patients had reduced identification rate and higher false-negative rates; accuracy improved with 3 or more SLNs
SN FNAC	2015	NAC	cN+	T0–T3, N1–2, M0 biopsy proven cN+ breast cancer	Post-NAC SLNB SLN identification rate was 87.6 % and FNR was 8.4 %
SINODAR-ONE	2022	Upfront surgery	cN– → pN1	Early-stage breast cancer and cN–	SLNB non-inferior to ALND for overall survival and recurrence
SENOMAC	2024	Upfront surgery	cN– → 1-2 macrometastatic SLNs	T1–T3, cN– patients with 1–2 macrometastatic SLNs on SLNB	Omission of ALND did not compromise DFS
NEOSENTI-TURK MF18	2025	NAC	cN+	cT1–T4, cN1–3 patients who underwent SLND or TAD	ALND is not required for patients with nodal pathologic complete response or limited residual disease post-NAC

cN–, clinically node-negative; ALND, axillary lymph node dissection; SLNB, sentinel lymph node biopsy; NAC, neoadjuvant chemotherapy; cN+, clinically node-positive; FNR, false-negative rate; SLN, sentinel lymph nodes; TAD, targeted axillary dissection

**Table 2:** Published trials evaluating omission of further axillary surgery or use of irradiation

Trial	Year	Initial treatment	Node status	Inclusion criteria	Comparison	Key findings
IBCSG 23-01	2013	Upfront surgery	cN– → low-burden SLN+	Primary invasive breast cancer	ALND vs no-ALND	Omission of ALND non-inferior to ALND in DFS and OS
AMAROS	2014	Upfront surgery	cN– → pN1	Primary invasive breast cancer and cN–	ALND vs axillary radiotherapy	ALND and axillary radiotherapy provide comparable locoregional control
OTOASOR	2017	Upfront surgery	cN– → pN1	Primary invasive breast cancer and cN–	ALND vs regional nodal irradiation	Regional nodal irradiation non-inferior to ALND in DFS, OS, and axillary recurrence
SOUND	2023	Upfront surgery	cN– (AUS–)	Invasive breast cancer (<2 cm) patients undergoing BCS and radiotherapy	SLNB vs no-SLNB	Omission of SLNB was non-inferior to SLNB in DFS
INSEMA	2024	Upfront surgery	cN– (AUS–)	Early-stage breast cancer patients undergoing BCS and radiotherapy	SLNB vs no-SLNB	Omission of SLNB was non-inferior to SLNB in DFS
ICARO	2024	NAC	cN0-3 → ypN0(i+)	Primary invasive breast cancer	ALND vs no further axillary surgery	ALND is not support in all patients with ypN0(i+)
NSABP B-51	2025	NAC	cN1 → ypN0	Stages I–III breast cancer patients and cN1	Regional nodal irradiation vs no regional nodal irradiation	Addition of adjuvant regional nodal irradiation did not decrease DFS or OS

cN–, clinically node-negative; SLN, sentinel lymph node; ALND, axillary lymph node dissection; DFS, disease-free survival; OS, overall survival; AUS, axillary ultrasound; BCS, breast-conserving surgery; SLNB, sentinel lymph node biopsy; NAC, neoadjuvant chemotherapy

**Table 3:** Ongoing/pending trials with primary oncolog0ic endpoints

Trial	Initial treatment	Question	Population	Primary endpoint	Results expected
Alliance A011202	NAC	Evaluate whether radiation to the undissected axilla is non-inferior to ALND with radiation to the regional nodes but not the dissected axilla	Stages I–III breast cancer patients with N1	Disease-free survival	Late 2020s
TAXIS	NAC or upfront surgery	Investigate whether tailored axillary surgery and axillary radiotherapy is non-inferior to ALND	Breast cancer patients with cN+	Disease-free survival	2036
EUBREAST-01	NAC	Can SLNB be omitted after pCR in the breast in response to NAC for TN and HER2+ breast cancer	Node-positive HER2+ or TN breast cancer patients who achieve pCR	Axillary recurrence-free survival	2027
ASLAN	NAC	Can SLNB be omitted after pCR in the breast in response to NAC for TN and HER2+ breast cancer	Node-positive HER2+ or TN breast cancer patients who achieve pCR	Disease-free survival	2028
NAUTILUS	Surgery	Can SLNB be omitted based on negative axillary imaging?	Early-stage breast cancer patients with cN–	Axillary recurrence	2027
AXSANA	NAC	Which post-NAC axillary approach provides adequate control?	cN+ breast cancer patients who underwent AUS after NAC	Disease-free survival	2030
POSNOC	Surgery	Is further axillary treatment necessary after limited SLN positivity?	Early-stage breast cancer patients with 1–2 positive SLNs	5-Year axillary recurrence	2027–2028
ADARNAT	NAC	Is axillary radiotherapy equivalent to ALND for residual nodal disease?	Breast cancer patients with residual nodal disease after NAC	Locoregional Control	Late 2020s
VENUS	Surgery	Can SLNB be omitted based negative clinical palpation and axillary ultrasound?	Early-stage breast cancer patients with cN– and negative AUS	Disease-free survival	2030
BOOG 2013-08	Surgery	Can SLNB be omitted in imaging-negative patients?	Early-stage breast cancer patients with cN–	Axillary recurrence	Late 2020s

cN+, clinically node-positive; NAC, neoadjuvant chemotherapy; ALND, axillary lymph node dissection; SLNB, sentinel lymph node biopsy; pCR, pathologic complete response; TN, triple-negative; HER2, human epidermal growth factor receptor 2; cN–, clinically node-negative; AUS, axillary ultrasound; SLN, sentinel lymph node

## The Present

### Standard Identification

Sentinel lymph node biopsy is based on the concept that lymphatic metastasis occurs in an orderly manner, with the SLN representing the first site of draining from the primary tumor. Accurate SLN identification therefore depends on reliable mapping techniques, including dyes and radiotracers.

Blue dyes are commonly used during SLNB to visualize lymphatic channels and stain the SLN. Isosulfan blue vital dye (Lymphazurin) is a triphenylmethane-based dye that was first adopted to provide clear intraoperative visualization during SLNB.^14^ In 1998, O’Hea et al.^40^ found that SLN identification with isosulfan blue vital dye alone is approximately 75 %. This dye can cause various adverse reactions, including blue hives, wheals and flares, and anaphylaxis in 1 % to 3 % of patients.^41^ Prophylactic intravenous hydrocortisone, diphenhydramine, and famotidine have been shown to reduce the severity of these adverse reactions.^42^

In 2002, concerns about isosulfan blue, including supply shortages and allergic reactions, prompted the use of methylene blue as an alternative. Simmons et al. reported a SLN identification rate of 92 % with methylene blue, and no adverse reactions were reported.^43^ Methylene blue dye is relatively inexpensive and widely available. However, local complications such as skin and fat necrosis have been reported, particularly when the dye is injected intradermally. In contrast to vital blue dyes, which typically are injected into subdermal lymphatics, methylene blue should be administered deeper within the breast parenchyma to reduce the risk of tissue injury.^44–46^

Indocyanine green (ICG) dye is a fluorescent dye that enables high-resolution, real-time visualization of lymphatic drainage under near-infrared light, improving SLN detection in patients with challenging or altered anatomy. Initial studies reported an identification rate of 73.8 %, but the rates in more recent studies exceed 90 %.^47–49^ In axillary staging, ICG dye has shown favorable trends compared with radioisotope and blue dye and often is used for postmastectomy SLNB, although its use in the United States remains limited.^50^ Practical limitations include a tendency to remove more SLNs, the need for a darkened field, and rapid subcutaneous spread that shortens operative timing. Additionally, because fluorescence does not permit visualization of the entire axilla without dissection, surgeons still must expose tissue planes to follow lymphatic channels, which may increase operative manipulation compared with probe-guided techniques.^51,52^

Radiotracers are injected into the peritumoral, intradermal, subareolar, periareolar, or subcutaneous tissue to map lymphatic drainage, allowing SLN detection using a gamma probe. The gold standard radiotracer is technetium-99m, which is used in several formulations including sulfur colloid, Nanocolloid, and tilmanocept.

Sulfur colloid is commonly administered by a nuclear medicine technician or radiologist, which can affect operative scheduling, although some centers allow perioperative injection by the surgeon. This formulation also is widely available and inexpensive, although it migrates more slowly through lymphatics than other technetium-99m formulations.^53^

Technetium-99m Nanocolloid migrates rapidly through lymphatics without sacrificing SLN retention time.^54^ Its particle size and stability make it well suited for accurate SLN mapping.^55^

Technetium-99m tilmanocept (Lymphoseek) demonstrates faster lymphatic migration and higher SLN retention than sulfur colloid or Nanocolloid.^54^ Patients also report less injection-site pain than with sulfur colloid. In states that allow intraoperative injection, LymphoSeek may be injected after the patient undergoes general anesthetic, immediately before SLNB.^56^

Many surgeons use dual-mapping, combining dye and radiotracer to improve SLNB accuracy. This approach leverages the strengths of both agents, yielding a lower FNR and a higher SLN identification rate.^57^ Its value is evident for post-NAC patients undergoing SLNB, as demonstrated in the ACOSOG Z1071 trial, in which dual-mapping reduced FNR from 12.6 % with a single agent to 10.8 %, supporting its routine use.^23^ As a result, dual-mapping has become standard practice in many centers for reliable axillary staging of NAC-treated patients.

When node-positive patients omit ALND, radiation oncologists often increase the use of regional nodal irradiation to maintain locoregional control. The AMAROS and MA.20 trials demonstrated comparable regional control between ALND and regional nodal irradiation, supporting its use as an effective alternative.^31,58^ This shift helps explain why local control remains high despite reduced axillary surgery.

The Choosing Wisely guidelines, endorsed by the Society of Surgical Oncology, recommends omitting SLNB for cN– patients 70 years old or older with early-stage, estrogen receptor (ER)-positive breast cancer. These guidelines are supported by numerous trials that demonstrated no increase in locoregional recurrence and improved quality of life when SLNB was omitted in this population.^59–61^ Despite these findings, adoption of SLNB omission has been limited. Goldhaber et al.^62^ reported that omission of SLNB for this patient population has increased only 20 % from 2012 to 2020, based on National Cancer Database (NCDB) data. Contributing factors may include patient preference for the reassurance of nodal staging and challenges navigating multidisciplinary cancer care.^63,64^

### Limitations of SLNB

Sentinel lymph node biopsy has revolutionized axillary management by markedly reducing the morbidity of ALND while maintaining non-inferior long-term outcomes. In addition, SLNB supports more personalized care by enabling surgeons to tailor axillary management to individual patient risk, anatomy, and treatment history. However, SLNB has important limitations, and its feasibility and diagnostic accuracy may be compromised in settings such as obesity and disrupted lymphatic drainage.

Obesity imposes technical challenges that may reduce the accuracy of SLNB. Body mass index (BMI) inversely influences the successful mapping of SLNs, particularly in patients 50 years old or older, and even when successful, fewer SLNs are retrieved.^65^ Excess adipose tissue can impede dye and radiotracer migration and reduce gamma probe sensitivity. These factors increase surgical time and operative difficulty, placing obese patients, who are already at elevated baseline risk for lymphedema, at further risk.^66^

Ipsilateral breast cancer recurrence presents additional challenges due to altered lymphatic drainage patterns in the axilla from previous axillary surgery and radiation, which may cause fibrosis and scarring.^67,68^ After previous axillary surgery, SLNB demonstrates lower identification rates (65–69 %), in part due to difficulty locating the SLN with radiotracers in disrupted pathways. Altered lymphatic anatomy also may lead to aberrant drainage and mapping to extra-axillary basins, including the internal mammary or contralateral axillary nodes, further complicating intraoperative identification.^68–71^ Although SLNB can remain accurate for carefully selected patients, identification rates may improve with adjunctive blue dye and meticulous preoperative planning.^68,69^

### Innovations

Recent advancements in SLNB have increased accuracy, reduced FNR, and increased the SLN identification rate. Key innovations that have emerged are TAD, which improves nodal staging accuracy after NAC, and new agents to improve FNR and SLN identification rates.

The TAD procedure ensures removal of the most clinically relevant lymph node, and the effects of chemotherapy can be accurately assessed. A systematic review by Swarnkar et al.^72^ reported an FNR of 5.16 % when TAD was performed after chemotherapy for cN+ patients. By accurately assessing residual nodal disease, TAD enables many patients to safely avoid cALND.

Localization of the clipped node can be achieved using several techniques including radioactive iodine-125 seed placement, radar reflector systems (e.g., SAVI Scout), magnetic seeds, or hookwire localization of the biopsied node. Radioactive iodine-125 seeds provide consistently high localization rates (97.6 %) with reliable intraoperative detection using a gamma probe but require nuclear medicine coordination and radiation safety infrastructure.^73^ Radar reflector systems demonstrate similarly high rates of localization (99.6 %) and retrieval (100 %) but require additional device cost and placement before surgery.^74^ Magnetic seed localization offers a non-radioactive alternative with retrieval rates of 99.13 % but requires magnetometer equipment.^75^ Hookwire localization has a retrieval rate of 99.63 %, offering same-day placement without specialized probes but is limited by potential wire displacement.^75^

Recent innovations in lymphatic mapping include superparamagnetic iron oxide (SPIO) tracers, which use iron oxide nanoparticles to identify sentinel nodes without radiation exposure.^76^ Magtrace (Endomagnetics Ltd., Cambridge, UK), a SPIO tracer, is injected similarly to conventional tracers and is detected with a handheld magnetometer, eliminating the need for nuclear medicine coordination. Additionally, Magtrace remains in lymphatic tissue for up to 30 days, allowing flexible scheduling.^77,78^ This prolonged retention has enabled delayed SLNB for patients with ductal carcinoma *in situ* (DCIS). The SentiNot study, found injection at the time of primary surgery for patients with a preoperative diagnosis of DCIS allowed accurate SLN retrieval weeks later if invasive carcinoma was identified on final pathology, thereby avoiding unnecessary upfront SLNB.^79^

## Future Directions

### Post-Surgical Quality of Life Outcomes

Although SLNB reduces morbidity compared with ALND, lymphedema still occurs for approximately 5 % of SLNB patients.^80^ Lymphaticovenous bypass (LVB), which anastomoses disrupted lymphatic channels to nearby venules, has emerged as a technique to restore lymphatic flow and reduce rates of lymphedema. Currently, LVB is being refined for ALND patients, and the rate of lymphedema has already been significantly reduced.^81,82^ A meta-analysis, which had a mean follow-up period of 22 months, found that LVB was associated with significant reductions in limb volume, cellulitis frequency, and compression use, supporting LVB as an effective intervention for secondary lymphedema.^83^

Persistent post-surgical pain (PPSP) occurs for approximately 29 % to 36 % of SLNB patients, commonly due to inadvertent injury to the intercostobrachial nerve.^84–86^ Prevention relies primarily on meticulous surgical technique and nerve preservation. Intraoperative nerve monitoring (IONM) may assist in preserving motor nerves such as the long thoracic and thoracodorsal nerves, but does not facilitate identification of purely sensory nerves such as the intercostobrachial nerve. In a randomized trial of modified radical mastectomy patients, IONM during ALND preserved postoperative electromyographic activity of the pectoralis major compared with dissection without monitoring, supporting its potential utility in breast cancer surgery.^87^

### Future Trials

The role of axillary radiation for patients with macroscopic axillary disease remains under active investigation. The Alliance A011202 trial is evaluating whether breast or chest wall radiation and regional nodal irradiation to levels 1 to 3 axillary nodes and supraclavicular fossa alone is non-inferior to ALND followed by breast or chest wall radiation and regional nodal irradiation to level 3 axillary nodes and supraclavicular fossa in patients with biopsy-proven cN+ disease who have residual disease after NAC.^88^ Similarly, the TAXIS phase 3 trial is comparing tailored axillary surgery (removal of palpable and radiographically abnormal axillary lymph nodes and SLNs) followed by axillary radiation to ALND for node-positive patients, either in the upfront surgery setting or after NAC. This trial aims to address the growing belief that axillary disease can be managed without ALND.^89^ Results from these trials, expected by 2030, may further redefine axillary management and expand the population eligible for SLNB in lieu of ALND.

Breast cancer patients who have SLNs with extracapsular extension (ECE) are at higher risk of additional nodal disease, and the extent of ECE is an important determinant of axillary tumor burden, with larger degrees of extension associated with increased nodal burden.^90^ The SENOMAC trial included post-NAC patients with ECE in one or two lymph nodes and demonstrated no compromise in 5-year recurrence rates among those who converted to node-negative status after NAC and omitted ALND.^29^ Although no subset analysis was performed on this group, the optimal management of ECE, particularly for patients with more extensive disease, remains to be fully defined.

The European Breast Cancer Research Association of Surgical Trialists 01 (EUBREAST-01) trial is evaluating the safety of omitting SLNB for patients with triple-negative or HER2-positive breast cancer who achieve pCR after NAC. These patients have the highest likelihood of pCR after NAC. This study aims to expand the eligibility criteria for SLNB omission, with results anticipated in 2027.^91^

The Avoid Axillary Sentinel Lymph Node Biopsy After Neoadjuvant Chemotherapy (ASLAN) trial is evaluating the omission SLNB for BCS patients ages 20 to 69 years with triple-negative or HER2+ breast cancer who achieve nodal pCR after NAC and receive adjuvant radiation. Final analysis is expected in 2028.^92^

The No Axillary Surgical Treatment for Lymph Node-Negative Patients After Ultra-Sonography (NAUTILUS) trial is randomizing cN– patients with early-stage breast cancer based on negative preoperative axillary imaging to SLNB or no axillary surgery. The primary endpoints include axillary recurrence and DFS. By directly testing imaging-guided omission of surgical nodal staging, NAUTILUS aims to determine whether SLNB can be safely avoided for selected patients. Completion is expected in 2027.^93^

The Axillary Surgical Techniques After Neoadjuvant Chemotherapy (AXSANA) trial is addressing uncertainty in post-NAC axillary management for cN+ patients who convert to node negativity. The cohort includes patients managed with SLNB, TAD, or ALND, with outcomes evaluated for axillary recurrence and DFS. Comparative analysis across these axillary approaches is intended to inform the safety of surgical de-escalation after NAC. Evaluation for study targets is expected in 2030.^94,95^

The Positive Sentinel Node: Omission of Axillary Clearance (POSNOC) trial is comparing adjuvant systemic therapy alone with adjuvant therapy plus axillary radiotherapy or axillary node clearance for women with early breast cancer and one or two SLN macrometastases. The primary endpoint is 5-year axillary recurrence, with secondary outcomes including DFS, OS, arm morbidity, and quality of life.^96^

The Axillary Dissection After Response to Neoadjuvant Therapy (ADARNAT) trial is evaluating axillary management of cN+ patients who have residual nodal disease after NAC identified by SLNB. Patients will be randomized to cALND or axillary irradiation and further stratified by SLN involvement. This trial addresses whether surgical clearance of the axilla is necessary for residual nodal disease after NAC or whether irradiation can provide equivalent oncologic outcomes.^97^

The Validation of Eliminating Nodal Surgery in Ultrasound-Negative Early-Stage Breast Cancer (VENUS) trial is comparing SLNB with no axillary surgery for patients with T1–T2 invasive breast cancer who are cN–. Patients are randomized to SLNB or omission of axillary surgery. The primary endpoint is DFS, with secondary endpoints compising OS and axillary recurrence.^98^

The Breast Oncology Onderzoek Groep (BOOG) 2013-08 trial is evaluating omission of SLNB for patients with early-stage, cN– breast cancer undergoing BCS. Patients with no clinical or imaging evidence of axillary nodal involvement are randomized to SLNB or no axillary surgery. The trial is designed to determine whether SLNB can be omitted without increasing regional recurrence.^99^

## Study Limitations

This review had several limitations. First, inclusion was restricted to literature published in English, potentially excluding relevant international studies. Second, although emerging technologies such as AI show promise, their cost-effectiveness, learning curve, and practical integration were not evaluated. Third, disparities in access to SLNB and surgical methods across health care systems were not evaluated, and feasibility may vary by resource availability.

## Conclusion

The evolution of axillary surgery reflects the broader transformation of breast cancer surgery toward biologically guided, morbidity-conscious treatment. Across upfront and post-NAC treatment settings and for patients with limited nodal involvement, trial data have shifted axillary surgery away from ALND toward a more selective approach, demonstrating that comparable oncologic outcomes can be achieved with less extensive surgery.

However, ALND is not obsolete. It remains necessary for patients with residual nodal disease after NAC, particularly in locally advanced cancers in which persistent tumor burden carries prognostic and therapeutic implications. The ALND procedure also continues to be indicated for inflammatory breast cancer, even in the setting of pCR given its aggressive biology and high risk of locoregional recurrence.

Important clinical gaps persist for patients with ECE, extensive nodal burden, or biologically aggressive disease. As axillary management continues to evolve, further refinement of response-guided and biologically tailored strategies will be essential to ensure that surgical de-escalation does not compromise oncologic precision or multidisciplinary decision-making.^100^
